# Genome-wide DNA methylation analysis pre- and post-lenalidomide treatment in patients with myelodysplastic syndrome with isolated deletion (5q)

**DOI:** 10.1007/s00277-021-04492-1

**Published:** 2021-04-27

**Authors:** Anna Hecht, Julia A. Meyer, Johann-Christoph Jann, Katja Sockel, Aristoteles Giagounidis, Katharina S. Götze, Anne Letsch, Detlef Haase, Richard F. Schlenk, Torsten Haferlach, Philippe Schafhausen, Gesine Bug, Michael Lübbert, Felicitas Thol, Guntram Büsche, Esther Schuler, Verena Nowak, Julia Obländer, Stephanie Fey, Nadine Müller, Georgia Metzgeroth, Wolf-Karsten Hofmann, Ulrich Germing, Florian Nolte, Mark Reinwald, Daniel Nowak

**Affiliations:** 1 Department of Hematology and Oncology, University Hospital Mannheim, Mannheim, Germany; 2 Department of Pediatrics, University of California San Francisco, San Francisco, CA USA; 3 Department of Hematology, University Hospital Dresden, Dresden, Germany; 4 Department of Hematology, Oncology and Palliative Care, Marien Hospital Duesseldorf, Duesseldorf, Germany; 5 Department of Internal Medicine, Technical University of Munich, Munich, Germany; 6 Department of Hematology and Oncology, Charité, Benjamin Franklin University, Berlin, Germany; 7 Clinics of Hematology and Medical Oncology, University Medicine Goettingen, Goettingen, Germany; 8 Department of Internal Medicine V, Heidelberg University Hospital, Heidelberg, Germany; 9 MLL Munich Leukemia Laboratory, Munich, Germany; 10 Department of Oncology, Hematology, BMT with section Pneumology, Hubertus Wald Cancer Center, University Medical Center Hamburg-Eppendorf, Hamburg, Germany; 11 Department of Internal Medicine, University Hospital Frankfurt, Frankfurt, Germany; 12 Internal Medicine, University Hospital Freiburg, Freiburg, Germany; 13 Department of Hematology, Hemostasis, Oncology, and Stem Cell Transplantation, Hannover Medical School, Hannover, Germany; 14 Hannover Medical School, Institute of Pathology, Hannover, Germany; 15 Department for Hematology, Oncology and Clinical Immunology, University Hospital, Heinrich-Heine-University, Duesseldorf, Germany; 16 Brandenburg Medical School Theodor Fontane, Brandenburg an der Havel, Germany

**Keywords:** Myelodysplastic syndromes, Deletion 5q, Lenalidomide, DNA methylation

## Abstract

Myelodysplastic syndrome (MDS) with isolated deletion of chromosome 5q (MDS del5q) is a distinct subtype of MDS with quite favorable prognosis and excellent response to treatment with lenalidomide. Still, a relevant percentage of patients do not respond to lenalidomide and even experience progression to acute myeloid leukemia (AML). In this study, we aimed to investigate whether global DNA methylation patterns could predict response to lenalidomide. Genome-wide DNA methylation analysis using Illumina 450k methylation arrays was performed on *n*=51 patients with MDS del5q who were uniformly treated with lenalidomide in a prospective multicenter trial of the German MDS study group. To study potential direct effects of lenalidomide on DNA methylation, 17 paired samples pre- and post-treatment were analyzed. Our results revealed no relevant effect of lenalidomide on methylation status. Furthermore, methylation patterns prior to therapy could not predict lenalidomide response. However, methylation clustering identified a group of patients with a trend towards inferior overall survival. These patients showed hypermethylation of several interesting target genes, including genes of relevant signaling pathways, potentially indicating the evaluation of novel therapeutic targets.

**Supplementary Information:**

The online version contains supplementary material available at 10.1007/s00277-021-04492-1.

## Introduction

Myelodysplastic syndrome (MDS) with isolated deletion of chromosome 5q (MDS del5q) is a biologically and phenotypically distinct subtype of MDS. Clinically, it is often characterized by severe macrocytic anemia and normal, or elevated, platelet counts. Patients with MDS del5q show a good overall survival and lower risk of evolution to acute myeloid leukemia (AML) compared to most MDS subtypes [1]. Furthermore, several large studies demonstrated an excellent response to the immunomodulatory drug lenalidomide for patients with MDS del5q [2–4].

The German MDS study group conducted a prospective, open-label, non-randomized, single-arm, multicenter, phase II trial to evaluate the safety of lenalidomide and identify patterns of disease progression [5]. Due to the strict inclusion criteria and rigid screening process, the study population was very homogenous. As expected, two-thirds of patients achieved sustained transfusion independence. However, progression to AML was observed in 15% of patients. This was not predictable by any clinical marker and raises the question of feasibility for potential combination therapies in addition to lenalidomide alone [5].

The mechanism of action of lenalidomide in MDS del5q has just recently been better understood [6]. As MDS del5q is hypothesized to be a disease of haploinsufficiency [7, 8], the specific sensitivity to lenalidomide is partially explained through its suppression of haplodeficient proteins by a cereblon-dependent increase of ubiquitination and degradation [9]. Additionally, lenalidomide has direct antiproliferative effects by inhibiting CDC25C and PP2A phosphatase levels which leads to G2/M-phase cell cycle arrest. It also induces T-cell and NK-cell proliferation and cytotoxic activity, resulting in immunomodulation [10–12].

Altered methylation of the genome is a known pathogenetic mechanism of MDS and has been shown to contribute to progression to AML [13–15]. Hypomethylating agents (HMA) are therefore widely used in the therapy of high-risk MDS and AML with myelodysplasia-related changes. HMAs are potential candidates for combination with lenalidomide in MDS del5q and are undergoing clinical evaluation. Whether lenalidomide itself has an effect on the methylation patterns of MDS patients is unknown. One recent study in multiple myeloma cell lines showed that DNA hypermethylation might be a mechanism of resistance to lenalidomide treatment [16]. This prompted us to evaluate the methylation status of patients with MDS del5q before and after uniform treatment with lenalidomide. We sought to answer the questions whether lenalidomide treatment alters methylation patterns in MDS, and whether methylation status can predict response to lenalidomide treatment and outcome of patients with MDS del5q.

## Methods

### Patients

Fifty-one patients who were diagnosed and uniformly treated within a German, multicenter, single-arm, open-label, phase II trial investigating the safety of lenalidomide in patients with MDS del5q were included in this study (MDS-LE-MON-5 study, EudraCT number: 2008-001866-10, www.clinicaltrials.gov: #NCT01081431). In addition to informed consent for the study according to the Declaration of Helsinki, patients had also consented to high-throughput molecular analyses. As specified by the inclusion criteria, all patients were classified as low to intermediate-1 risk according to IPSS with a bone marrow blast cell count below 5%. The cohort consisted of 9 male and 42 female patients with a median age of 69 years (range 40–87 years) at time of enrollment. Details on the study protocol and results have been previously published [5]. In short, all patients uniformly received lenalidomide at a dose of 10 mg daily for 21 days of every 28-day cycle. Patients received a median of 13 cycles (range: 1–49 cycles). In 20 patients (39%), treatment had to be ended due to toxicity or incompliance before the completion of 6 cycles.

Additionally, for 17 of the 51 patients, we obtained follow-up bone marrow samples 4–6 months after the start of lenalidomide treatment.

### HumanMethylation450 bead array sample and data processing

Mononuclear cells from patients’ bone marrow were isolated using Ficoll density gradient centrifugation and DNA was extracted using the Allprep Kit (Qiagen, Hilden, Germany). Following bisulfite conversion, genome-wide DNA methylation analysis was performed using the HumanMethylation450 BeadChip (Illumina, San Diego, USA) according to the manufacturer’s protocol.

Raw data was processed using the minfi R package [17] as previously described [18]. In short, the functions preprocessNoob, maptoGenome, and ratioConvert were utilized to produce beta-values and *M*-values. Probes that mapped either to regions with known germline polymorphisms (Illumina supplementary SNP list v.1.2 update table v.3), to multiple genomic loci [19], or to the sex chromosomes were filtered out. This left 295,926 probes for primary analysis.

### Hierarchical clustering for sample classification

For the comparison of 17 paired patient samples (pre-lenalidomide and 4–6 months post-treatment), beta-values were hierarchically clustered in both directions (samples and probes) using an unsupervised approach based on Ward’s minimum distance method. Due to the uniformity of the cohorts’ methylation patterns, probes with standard deviations (SD) greater than 0.20 across samples were chosen for clustering. This resulted in 934 probes. For the analysis of all patients at the start of the study, unsupervised hierarchical clustering was repeated using the whole cohort of 51 pre-treatment samples. Here, a cutoff of SD of greater than 0.20 resulted in 1629 probes for clustering.

### Pathway analyses

Probes within promoter regions were associated with the corresponding genes based on the Genecode v19 gene annotations [18]. The most significantly differentially methylated probes between patients clustering in different clusters were identified by multiple *t*-tests on the methylation *M*-values. A false discovery rate approach with a *q*-value set to <0.05 was used following the two-stage step-up method by Benjamini et al. [20]. Pathway analysis was performed on the genes associated with the identified probes. KEGG analysis was performed using KEGG Mapper (http://www.genome.jp/kegg/mapper.html). Gene ontology analysis and chromosomal mapping were performed using the GATHER tool (http://changlab.uth.tmc.edu/gather/).

### Statistical analysis

Differences in clinical features between patients clustering in different clusters were tested for significance using the chi-squared test for categorical variables and the Mann-Whitney *U* test for continuous variables. Overall survival (OS) was defined as time from start of lenalidomide treatment to death from any cause. Progression-free survival (PFS) was defined as time to progression or death from any cause. Both were estimated by the Kaplan-Meier method using a log-rank test for significance. Median follow-up time was estimated using the reverse Kaplan-Meier method. The significance level for all tests was a *p*-value <0.05. All calculations were performed using Microsoft Excel (v16.16.3), GraphPad Prism software (v8.0), and R (v4.0.2).

## Results

### Genome-wide methylation patterns are stable during lenalidomide treatment

**Fig. 1:**
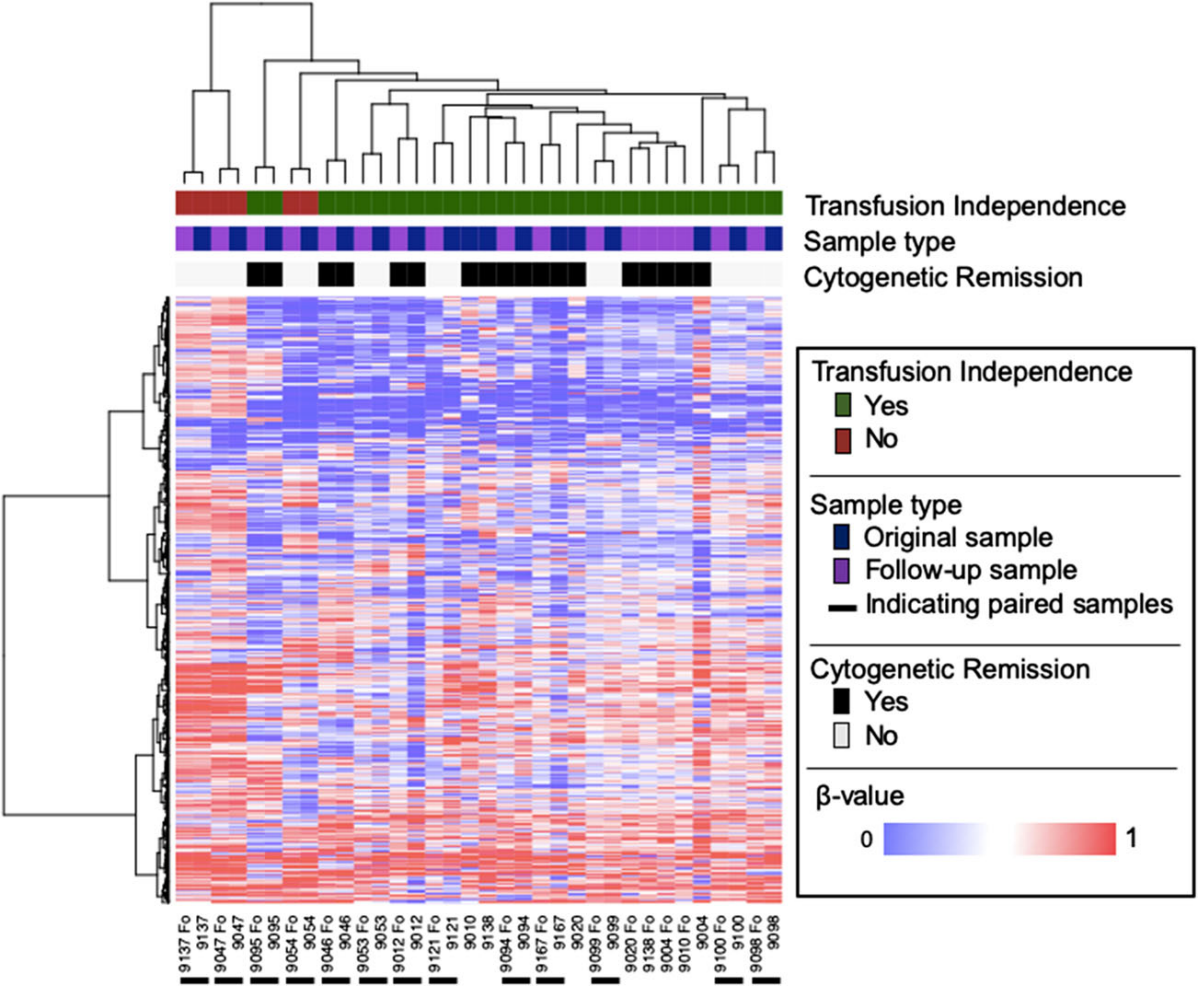
Unsupervised clustering between pre- and post-lenalidomide samples shows no relevant difference of methylation after treatment. Patients are displayed on the X axis and the 934 most variable CpG sites are displayed on the Y axis. Information on transfusion independence, sample type, and cytogenetic remission is shown at the top of the figure

To elucidate whether lenalidomide treatment leads to changes in global methylation patterns, we generated Illumina 450k methylation data using bone marrow mononuclear cells from 17 patients of the LE-MON-5 study at the start of the study and 4–6 months after the start of lenalidomide treatment. For clustering, we initially wanted to use only probes with a differential standard deviation (SD) above 0.25 among patient samples. However, this resulted in a very low number of probes (111 probes), demonstrating that the methylation pattern across the cohort seemed to be very homogenous. As a consequence, probes with SD above 0.20 were used for this analysis (934 probes). We performed unsupervised hierarchical clustering on the 34 patient samples (17 pairs of samples prior to and post-lenalidomide treatment). In the majority of cases (13 out of 17 sample pairs), both samples from the same patient clustered immediately together. This was observed equally for patients who achieved a cytogenetic remission and for patients who did not. No relevant changes in methylation could be observed between samples before and after lenalidomide treatment (Fig. 1).

### MDS patients with del5q have two distinct clusters of methylation

**Fig. 2:**
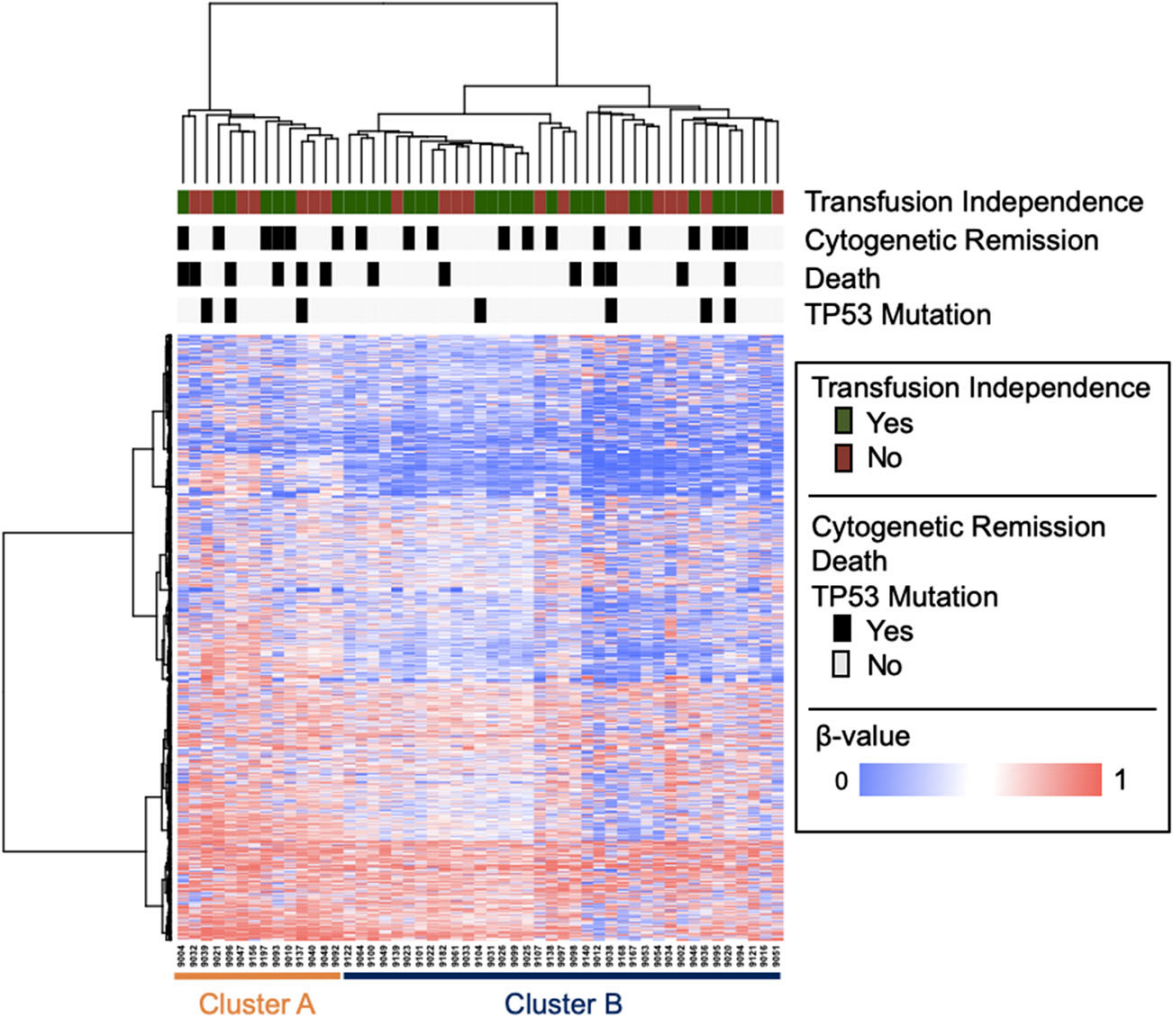
Unsupervised clustering of 51 patient bone marrow samples prior to lenalidomide treatment reveals two distinct clusters. Patients are displayed on the X axis and the 1629 most variable CpG sites are displayed on the Y axis. Information on transfusion independence, cytogenetic remission, death, and TP53 mutation status is shown at the top of the figure

Next, we wanted to investigate whether the methylation status present before the start of lenalidomide treatment could predict response to the drug. Therefore, we generated Illumina 450k methylation data for an additional 34 MDS patients with del5q at the start of the study. Again, the expanded cohort of 51 patients overall proved to have a relatively homogenous methylation pattern. A more stringent cutoff of probes with a SD above 0.25 resulted in only 172 probes for clustering. Accordingly, probes with SD above 0.20 were used for the analysis. Unsupervised clustering based on the 1629 most variably methylated CpGs showed a clear distinction of patients based on their methylation pattern before the start of treatment. Patients were divided into two main clusters: cluster A and cluster B (Fig. 2). The 1629 CpGs informing the clustering were associated with 961 genes (Supplemental Table 1). The gene set was most enriched for genes in signaling pathways (including PI3K-Akt-, MAPK-, cAMP-, and calcium signaling), metabolic pathways, and cell-cell adhesion. There was a significant enrichment of genes mapping to chromosome 5q31 (34 genes, *p*<0.0001).

### Comparison of patient characteristics between methylation clusters

Baseline characteristics of patients in both clusters at the start of the study are shown in Table 1. Despite filtering out probes that mapped to either sex chromosome, there was a significantly higher fraction of male patients in cluster A compared to cluster B (36% in cluster A (5/14 patients) vs. 11% in cluster B (4/37 patients); *p*=0.04). Apart from that, patients in both clusters showed similar baseline characteristics such as age, blood counts, or bone marrow blast percentage. There was no significant difference in the median number of lenalidomide cycles patients had received or in the percentage of patients in both groups that responded to therapy with lenalidomide, showed cytogenetic remission, or experienced disease progression. The number of patients with mutations in *TP53* was higher in cluster A, but this difference did not reach statistical significance. Furthermore, there was no significant difference in the number of patients with intermediate-1 vs. low-risk (including very low risk) IPSS(-R) scores. However, there was a trend towards a difference in the number of deaths between the cohorts: the proportion of patients who died in cluster A was 43% (6/14 patients) compared to 19% in cluster B (7/37 patients, *p*=0.08).

**Table 1:** Comparison of clinical parameters of patients according to methylation clusters

	Cluster A	Cluster B	*p*-value
	*n*=14	*n*=37	
Median age, y	68	70	0.76
Range	55–79	40–87	
Gender, male/female	5/9	4/33	0.04 (*)
Median Hb, g/dl	8.9	9	0.61
Range	6.3–11	6.4–11	
Median ANC, /μl	1972	1782	0.41
Range	420–11,299	890–11,200	
Median PLT, /μl	154	293	0.28
Range	70–674	69–1829	
Median ferritin, ng/ml	1281	991	0.56
Range	316–5880	255–6471	
Median BM blasts, %	2.5	2	0.73
Range	0–5	0–4	
*TP53*mut, %	21%	11%	0.29
*TP53* WT, %	79%	89%	
Median cycles of lenalidomide, *n*	15	13	0.42
Range	1–33	1–49	
Transfusion independency, %	50%	65%	0.33
Cytogenetic response, %	43%	20%	0.37
Progression, %	29%	14%	0.21
Death, %	43%	19%	0.08
IPSS#			0.57
Low	8	23	
int-1	6	12	
IPSS-R#			0.21
Very low	2	4	
Low	9	28	
int	3	3	

*Abbreviations*: *Hb*, hemoglobin; *ANC*, absolute neutrophil count; *PLT*, platelets; *BM*, bone marrow; *TP53mut/WT*, mutated or wild-type version of *TP53*; *IPSS(-R)*, international prognostic scoring system (revised)

^#^Information on 2 patients missing

### Methylation pattern is associated with a trend towards inferior outcome

**Fig. 3:**
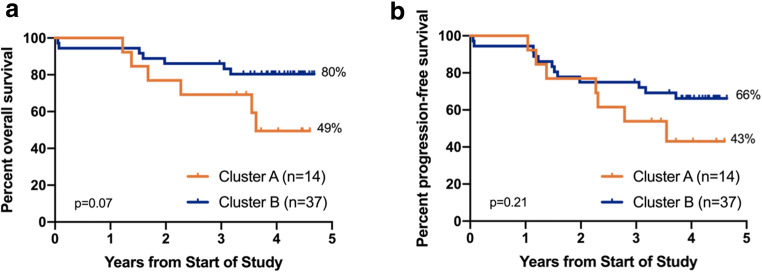
Overall survival (**a**) and progression-free survival (**b**) of the 51 patients were estimated using the Kaplan-Meier method. Patients are differentiated by clusters A and B (compare to Fig. 2)

Estimate of overall survival using the Kaplan-Meier method showed a trend towards inferior survival for patients clustering in cluster A. Estimated survival of these patients after a median follow-up of 4.2 years (range 0.1–4.7 years) was 49.5% (95% CI: 20–74%, median survival: 3.6 years) compared to 80.4% (95% CI: 63–90%, median not reached) for patients clustering in cluster B (Fig. 3a; *p*=0.07). This difference in survival could not be explained by the significantly higher number of male patients in cluster A as there was no difference in survival between male and female patients (Supplemental Figure 1). In contrast, there was no significant difference in PFS between the two cluster cohorts: PFS was 43.1% for patients in cluster A (95% CI, 16–68%) and 66.2% for patients in cluster B (95% CI, 48–79%; *p*=0.21; Fig. 3b).

### Methylation clustering differences are not driven by TP53 mutational status

To evaluate whether the differences in methylation and survival between the clusters were driven by the higher number of *TP53* mutations in cluster A, we repeated the unsupervised clustering for all patients excluding patients with *TP53* mutations. Based on the resulting 1534 most variably methylated CpGs, *TP53* wild-type patients clustered into the same two clusters as before (Supplemental Figure 2- compare to Figure 2). Additionally, after removing patients with *TP53* mutations, there was still a trend towards inferior survival of patients clustering in cluster A (Supplemental Figure 3).

### Pathway analyses of differentially methylated genes

To determine which CpGs were significantly differentially methylated between clusters A and B, multiple *t*-testing was executed on sample *M*-values with an accepted false discovery rate of 5% (*q*-value <0.05). This resulted in a list of 523 significantly differentially methylated CpGs which were associated with 319 genes (Supplemental Table 2). The vast majority (95%) of these CpGs were hypermethylated in cluster A compared to cluster B. Chromosomal mapping again identified a significant enrichment of genes mapping to chromosomal location 5q31 (Table 2). KEGG pathway analysis of the 319 genes revealed a significant enrichment in several pathways associated with cancer, including MAPK- and PI3K-Akt-signaling, as well as cancer-associated microRNAs (Table 3 and Table 4). Additionally, two members of the WNT-pathway (*Frizzled class receptor* 5, *FZD5* and *Leucine Rich Repeat Containing G Protein-Coupled Receptor 6*, *LGR6*) were found to be significantly different between the clusters. Further interesting candidate genes previously associated with myeloid neoplasms found in the analysis, included *WT1* (*Wilms’ tumor 1*), *PITX2* (*Paired like homeodomain 2*), *PDGFRA* (*Platelet Derived Growth Factor Receptor Alpha*), *BMP4* (*Bone Morphogenetic Protein 4*), *ERBB2* (*Erb-B2 Receptor Tyrosine Kinase 2*), and several members of the *HOX* (*Homeobox*) transcription factor gene family.

**Table 2:** Chromosome mapping of significantly differentially methylated genes

Chromosome location	# of genes	*p*-value
5q31	6	0.004
4q25	3	0.009
19 cen	1	0.02

**Table 3:** Top hits of KEGG pathway enrichment analysis (number of genes)

hsa01100 Metabolic pathways - Homo sapiens (human) (9) hsa05200 Pathways in cancer - Homo sapiens (human) (7) hsa05206 MicroRNAs in cancer - Homo sapiens (human) (6) hsa04010 MAPK signaling pathway - Homo sapiens (human) (6) hsa04151 PI3K-Akt signaling pathway - Homo sapiens (human) (5) hsa04530 Tight junction - Homo sapiens (human) (5) hsa05205 Proteoglycans in cancer - Homo sapiens (human) (5) hsa04310 Wnt signaling pathway - Homo sapiens (human) (2)

**Table 4:** Top hits of gene ontology analysis

Gene ontology	# of genes	*p*-value
GO:0007275 [2]: development	36	< 0.0001
GO:0009653 [3]: morphogenesis	23	0.002
GO:0007156 [6]: homophilic cell adhesion	6	0.006
GO:0007389 [3]: pattern specification	3	0.007
GO:0009887 [4]: organogenesis	18	0.01
GO:0048513 [3]: organ development	18	0.01
GO:0017144 [5]: drug metabolism	2	0.01

## Discussion

We present a comprehensive genome-wide DNA methylation analysis on 51 MDS del5q patients. This represents the largest cohort of MDS del5q patients analyzed for methylation so far. In an earlier analysis by Zhou et al. [15] on 20 MDS patients, only one patient with del5q was included and, interestingly, this patient clustered with the healthy controls rather than the other MDS subtypes. In a larger, more recent study, Reilly et al. [21] analyzed 10 patients with del5q in their cohort of 141 MDS patients and found genes in the respective cluster were mostly hypermethylated; especially, the genes *WT1*, *NBEA* (*Neurobeachin*), and *AP1M2* (*Adaptor Related Protein Complex 1 Subunit Mu 2*) were significantly differentially methylated. All three genes were also found to be significantly differentially methylated in our cohort. Of note, *WT1* hypermethylation was associated with inferior survival in their study.

The main focus of the present study was the comparison of global methylation prior to and after treatment with lenalidomide. For this, we were able to obtain paired samples from 17 patients. As expected in this very homogenous patient population, we found a very homogenous methylation pattern across patients before the start of therapy. Comparing pre- and post-treatment samples, we found no relevant changes in methylation. This observation was independent of whether patients had achieved a cytogenetic remission. On one hand, this highlights the consistency of our sample and data processing. On the other hand, it shows that methylation patterns seemed to be mostly independent of the size of the del5q clone and lenalidomide treatment had little effect on the genome-wide methylation status of the patients’ bone marrow cells in this study. Lenalidomide does not seem to exert its observed effect by inducing DNA methylation changes.

Despite the fact that the study population of the LE-MON-5 study was very homogenous due to the rigid screening process, there was still one third of patients who did not respond to lenalidomide treatment. Therefore, we next wanted to explore whether differences in the baseline methylation patterns between patients could help predict the response to lenalidomide treatment. Unsupervised hierarchical clustering showed a clear distinction of patients into two main clusters. Even though the percentage of responders in cluster B was higher than in cluster A, this difference was not significant and the methylation status not predictive of response. We additionally performed supervised clustering between responders and non-responders to lenalidomide treatment. The resulting heatmap showed no differences in methylation patterns. This was further confirmed by multiple *t*-testing on the sample *M*-values, which resulted in zero significantly differently methylated CpGs between responders and non-responders (data not shown). In contrast, there was a clear trend towards inferior survival for patients in cluster A. One potential explanation could be the higher number of patients harboring a mutation in *TP53*, which has previously been associated with inferior outcome in MDS del5q [22]. However, even after excluding patients with *TP53* mutations from the analysis, the remaining patients still clustered into the same two clusters and there was still a trend towards inferior survival for patients in cluster A independent of *TP53* mutations. Only half of the deaths in cluster A were due to progressive disease (only one of them in a *TP53* mutant patient; the other three deaths were due to cardiac disease or unknown reasons) and PFS was in fact not significantly different between the two clusters.

To evaluate whether the methylation differences between the clusters were influenced by the significantly higher number of male patients in cluster A, we compared our data to previously published data on the influence of gender on the methylation of autosomes [23]. In contrast to the reported finding that male gender is associated with less methylation, cluster A was predominantly composed of hypermethylated CpGs. Additionally, none of the genes reported to be significantly altered by gender and only a very small fraction (around 5%) of the genes considered as potentially altered by gender in the study by Liu et al. [23] were among our most differentially methylated CpGs. Therefore, we conclude that the methylation differences observed between the clusters cannot be simply explained by gender distribution.

Analyzing the significantly differentially methylated genes leading to the clusters, we found that the majority of genes were hypermethylated in cluster A compared to cluster B. Among those genes, there were some interesting candidates whose aberrant methylation had been associated with inferior outcome in MDS in prior studies, e.g., *WT1* [21], *DLX6* (*Distal-Less Homeobox 6*) [24], members of the *PI3K-Akt-mTOR* and *MAPK* pathways [14] and, importantly, members of the *Wnt* signaling pathway. There have been several reports of a role of *Wnt* signaling in the pathogenesis of MDS del5q and its potential as a therapeutic target [25, 26]. Initially, it was observed by Liu et al. [27] that *CTNNA1* (*Catenin Alpha 1*), a tumor suppressor gene encoded on chromosome 5q31, was expressed at lower levels in MDS del5q due to suppressed expression of the second copy by methylation of the gene promotor. Since then, several studies have reported aberrant methylation of *Wnt* family members in MDS including del5q underlining the potentially important role of the regulation of this pathway [14, 28–30].

In summary, utilizing a homogenous, uniformly treated cohort of patients with MDS del5q, we could show that lenalidomide treatment does not have a relevant impact on genome-wide DNA methylation in MDS del5q. Still, before start of treatment, methylation analysis was able to identify a distinct subgroup of patients (27%) with a trend towards inferior overall survival but not inferior progression-free survival. This group showed hypermethylation in a number of interesting cancer-associated genes. Whether this translates into a potential benefit through the use of HMAs is a question this study cannot answer and that needs to be further evaluated in lenalidomide + HMA combination studies. Independently, our study provides additional indications towards the potential evaluation of *Wnt* signaling as a therapeutic target in MDS del5q.

## Supplementary information

ESM 1 (PDF 296 kb)

Supplementary Table 1 (XLSX 68 kb)

Supplementary Table 2 (XLSX 29 kb)

## Data Availability

The datasets generated during and analyzed during the current study are available from the corresponding author on reasonable request.

## Code availability

Code used for the analysis of data in this study is available from the corresponding author on reasonable request.
