# Supplementary material for: Genome-wide DNA methylation analysis pre- and post-lenalidomide treatment in patients with myelodysplastic syndrome with isolated deletion (5q)
Source: Ann Hematol. 2021 Apr 27;100(6):1463–71. doi: 10.1007/s00277-021-04492-1 (PMC8116243; doi:10.1007/s00277-021-04492-1)

Supplemental Material for Manuscript "Genome-wide DNA methylation analysis pre- and post-lenalidomide treatment in patients with myelodysplastic syndrome with isolated deletion (5q): results from a prospective multicenter study of the German MDS study group", Annals of Hematology

Anna Hecht[1], Julia A. Meyer[2], Johann-Christoph Jann[1], Katja Sockel[3], Aristoteles Giagounidis[4], Katharina S. Götze[5], Anne Letsch[6], Detlef Haase[7], Richard F. Schlenk[8], Torsten Haferlach[9], Philippe Schafhausen[10], Gesine Bug[11], Michael Lübbert[12], Felicitas Thol[13], Guntram Büsche[14], Esther Schuler[15], Verena Nowak[1], Julia Obländer[1], Stephanie Fey[1], Nadine Müller[1], Georgia Metzgeroth[1], Wolf-Karsten Hofmann[1], Ulrich Germing[15], Florian Nolte[1], Mark Reinwald[16] and Daniel Nowak[1].

[1]Department of Hematology and Oncology, University Hospital Mannheim, Mannheim, Germany; [2]Department of Pediatrics, University of California San Francisco, San Francisco, USA; [3]Department of Hematology, University Hospital Dresden, Dresden, Germany; [4]Department of Hematology, Oncology and Palliative Care, Marien Hospital Duesseldorf, Duesseldorf, Germany; [5]Department of Internal Medicine, Technical University of Munich, Munich, Germany; [6]Department of Hematology and Oncology, Charité, Benjamin Franklin University, Berlin, Germany; [7]University Medicine Goettingen, Clinics of Hematology and Medical Oncology, Goettingen, Germany; [8]Department of Internal Medicine V, Heidelberg University Hospital, Germany; [9]MLL Munich Leukemia Laboratory, Munich, Germany; [10]Department of Oncology, Hematology, BMT with section Pneumology, Hubertus Wald Cancer Center, University Medical Center Hamburg-Eppendorf, Hamburg, Germany; [11]Department of Internal Medicine, University Hospital Frankfurt, Frankfurt, Germany; [12]University Hospital Freiburg, Internal Medicine, Freiburg, Germany; [13]Department of Hematology, Hemostasis, Oncology, and Stem Cell Transplantation, Hannover Medical School, Hannover, Germany; [14]Hannover Medical School, Institute of Pathology, Hannover, Germany; [15]Department for Hematology, Oncology and Clinical Immunology, University Hospital, Heinrich-Heine-University, Duesseldorf, Germany; [16]Brandenburg Medical School Theodor Fontane, Brandenburg an der Havel, Germany.

Corresponding author: Anna Hecht, MD, E-Mail: anna.hecht@medma.uni-heidelberg.de

Supplemental Figure 1: Overall survival of the 51 patients depending on gender estimated using the Kaplan-Meier method.

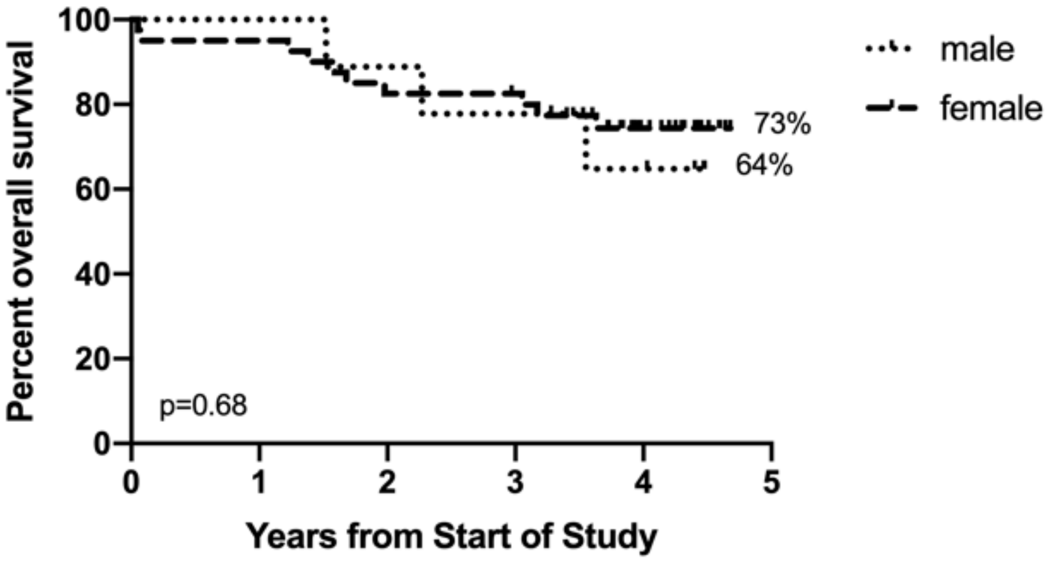

Supplemental Figure 2: Unsupervised clustering of 44 patient bone marrow samples prior to lenalidomide treatment. Patients with mutations in TP53 were excluded from analysis. After exclusion of TP53mut patients, each of the TP53wt patients still clusters in the same way as before when analyzing the whole cohort. Patients are displayed on the X axis and the 1534 most variable CpG sites are displayed on the Y axis. Information on transfusion independence, cytogenetic remission and death is shown at the top of the figure.

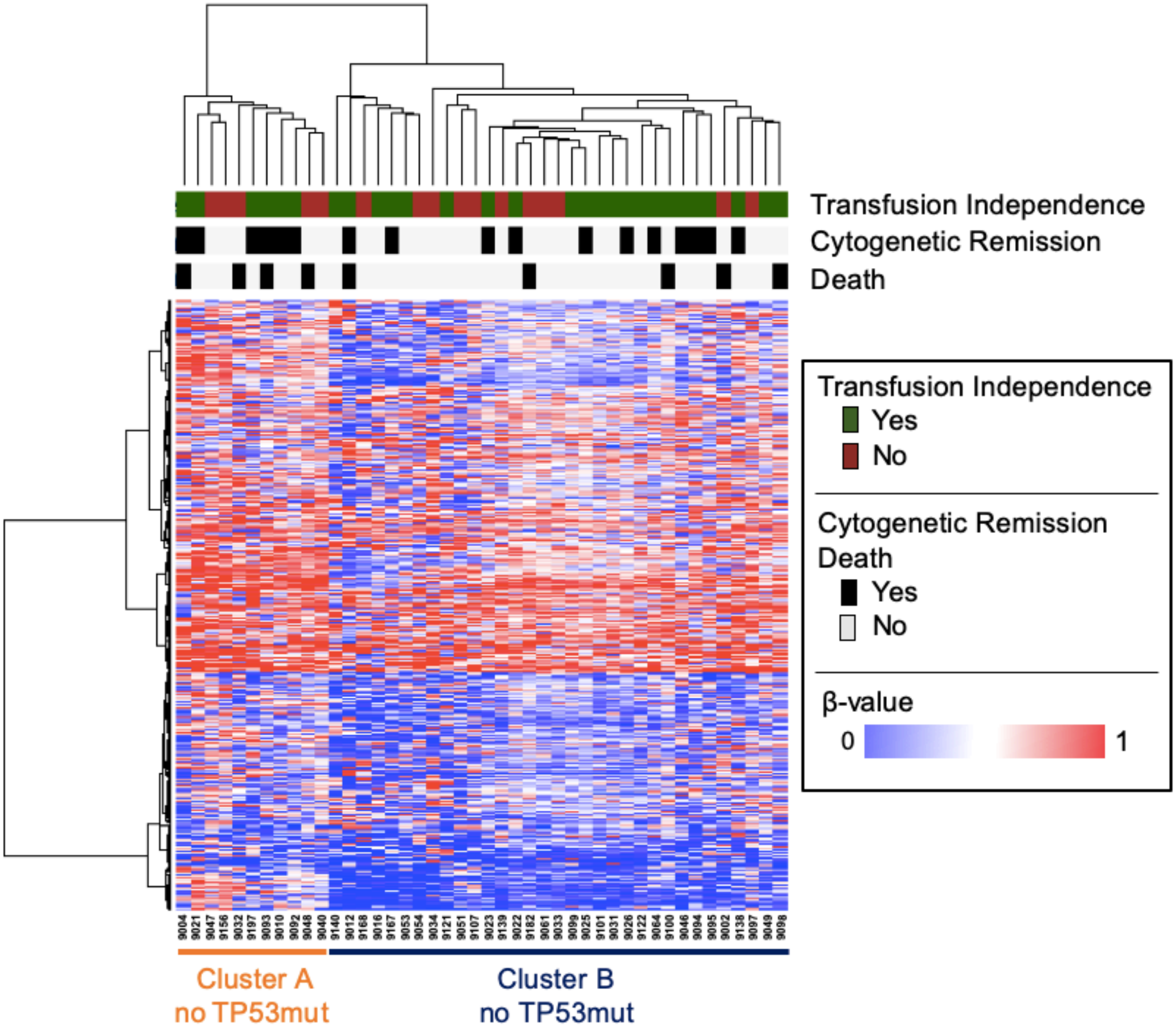

Supplemental Figure 3. Overall survival of the 44 patients without TP53 mutations was estimated using the Kaplan-Meier method. Patients are differentiated by Cluster A and B (compare to Supplemental Figure 2).

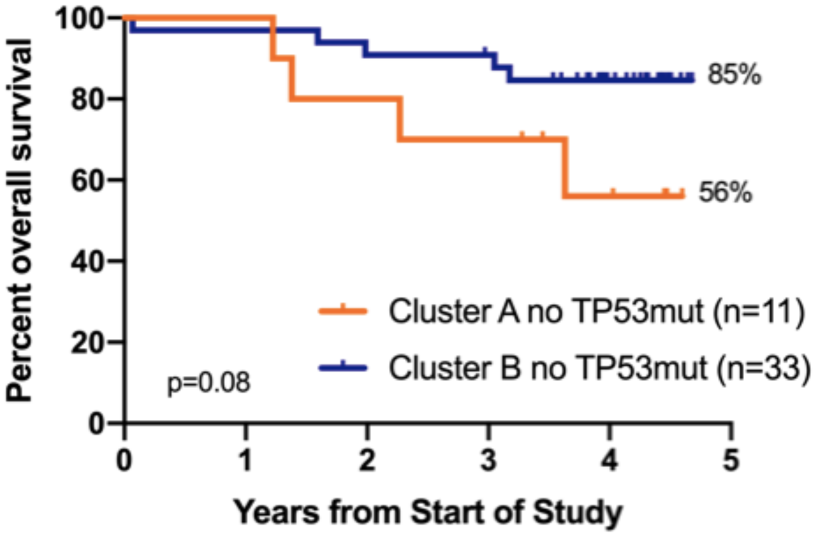

Supplement: Supplementary file 1 — (PDF 296 kb) [file 277_2021_4492_MOESM1_ESM.pdf]
